# Predictors of adverse drug reaction among adult HIV-infected patients on antiretroviral therapy in government hospitals of Kaffa Zone, Ethiopia; November 2018: a retrospective cohort

**DOI:** 10.11604/pamj.2021.38.181.19915

**Published:** 2021-02-17

**Authors:** Ayanaw Ambachew Mitkie, Fanuel Belayneh Bekele, Alemu Tamiso Debiso

**Affiliations:** 1 Chena Woreda Health Office, Kaffa Zone, Ethiopia,; 2 School of Public Health, College of Medicine and Health, Hawassa University, Hawassa, Ethiopia

**Keywords:** Antiretroviral therapy, Ethiopia, HIV/AIDS, adverse drug reaction

## Abstract

**Introduction:**

incidence of adverse drug reactions (ADR) associated with antiretroviral therapy (ART) was higher in developing countries. In two teaching hospital in Ethiopia: Debremarkose 23% and Yirgalem 73.2% of study participants reported at least one ADR. Since there was limited information about ADR in the study area; we aimed to determine its incidence-rate and predictors.

**Methods:**

we conducted retrospective cohort study using medical records of HIV-infected patients enrolled on ART between 2006 and 2017 in government hospitals of Ethiopia. ADR was defined as report of at least one unwanted response to ART. We run descriptive and cox regression analysis (CRA).

**Results:**

incidence-rate of ADR was 4.1 per 100 person-years (py). Hazards of ADR among patients living at rural was almost two times than at urban; [Adjusted hazard ratio (AHR): 1.94(95% (CI): 1.18, 3.20)]. Stavudine (D4T)-Lamivudine (3TC)-Nevirapine (NVP) had about two times [AHR: 1.78(95%CI: 1.03, 3.08)], Zidovudine(AZT)-3TC-NVP had about two times [AHR: 2.34 (95%CI: 1.20, 4.57)], D4T-3TC-Efaviranze(EFV) had about three times [AHR: 2.86(95%CI: 1.38, 5.95)] and AZT-3TC-EFV had about two times [AHR: 2.16(95%CI: 1.21,3.90)] hazards of ADR than Tenofovir(TDF) based regimens. Being WHO clinical stage III had about two times hazard of ADR [AHR: 2.46 (95%CI: 1.22, 4.95)] and IV had about four times hazard of ADR [AHR: 4.32 (95%CI: 1.88, 9.93)] than stage I.

**Conclusion:**

risk of ADR was higher among adult HIV-infected patients on ART living in rural, WHO clinical stage III and IV, and patients on AZT and D4T based regimen. AZT should not be given as an alternative treatment, increase access of TDF regimens.

## Introduction

Since the start of acquired immune deficiency syndrome epidemic, more than 70 million people infected and 35 million died worldwide [1]. Sub-Saharan Africa remains the most severely affected region, with nearly 1 in every 25 adult (4.2%) living with HIV [2]. Treatment for HIV involves multiple drugs, each with own side effects [3]. ADR is defined as; a noxious, unwanted, unpredictable and unintended response to drug taken correctly [4].The spectrum of ADRs associated with ART varies between developed and developing countries, and typically ranges from 11-35.9% reported in developing countries [5]. In two studies in Ethiopia, 23% and 73.2% the study participants in two teaching hospitals (Debremarkose and Yirgalem respectively) reported at least one ADR [6, 7]. Currently, TDF and AZT are given with other regimens for initiation of care for HIV-infected patients in Ethiopia; AZT alone is considered as an alternative. Teaching hospitals (tertiary hospitals) provide high-level specialized care, and might have different population compared with primary public governmental hospitals. To determine incidence and predictors of ADRs in Ethiopia, we evaluated data from government hospitals in Kaffa Zone, Ethiopia during July 2006 and August 2017.

## Methods

**Study setting:** this study was conducted in Gebretsadik Shawo Memorial General Hospital (GSMGH) and Wacha Melese Zenawi Memorial Primary Hospital (WMZMPH) government hospitals of Kaffa Zone between June and August 2018. GSMGH and WMZMPH are the only general and primary hospitals in the zone serves as a referral site. ART service was started in both hospitals in 2006.

**Study populations and design:** we conducted a retrospective cohort study. We reviewed all baseline and follow-up clinical records of HIV-infected adult patients aged > 15 years who started ART between July 2006 and August 2017. All patients on ART with complete baseline and at least single follow-up after enrolment were included; duplicated and illegible data were excluded.

**Sample size determination and sampling technique:** we calculated sample size for incidence and predictors of ADR using EPI info V.7. We used a single population formula to calculate the incidence of ADR, using a confidence level of 95%, a margin of error 5% and incidence of ADR 23% based on the study in Debremarkose hospital. Using this estimations, the sample size was 203 [6]. We compared this with a sample size for power with 80% power, 95% confidence, a 5% margin of error, and a relative risk of ADR based on educational level of 1.5, for a sample 530 [6]. During our study period, 797 patients registered on ART. Of those, 592 clinical records had complete baseline data with at least single follow-up, after enrollment for ART had been included. Dependent variables included incidence of ADR and ADR-free survival time. Independent variables included, variables measured during initiation of HIV-infected adult patients for ART; socio-demography, baseline CD4 count, body mass index, clinical stages, functionality status of the patient, comorbidity, ART treatment type and eligibility criteria for ART.

**Data collection method and tool:** data were collected using standard data abstraction sheet; informed by reviewing previous literature as well as available ART intake and follow-up forms. Data were collected by ART trained clinical nurses and public health officers. A three-day orientation was given for supervisor and data collector.

**Data processing and analysis:**data were checked, coded, cleaned and entered in to Epi-info version 7.2.2, then exported and analyzed by SPSS version 20. Variables with P-value (P < 0.25) on the bi-variable CRA were selected as candidates for multivariable CRA. On multivariable CRA, variable with P<0.05 declared as statistically significant predictor of incidence of ADR. We used a Kaplan-Meier curve to assess the cumulative survival. We summarized the data using mean, median and AHR, and presented with graph, table and text.

### Operational definitions

**ADR:** a new case of unfavorable condition reported at least once on HIV-infected adult patients after enrollment on ART. ADR included anemia, skin rash, peripheral neuropathy, abdominal pain, lipodystrophy, jaundice, nightmare and nephrites; censored: no ADR reported during follow-up period after initiation on ART. Starting time: time when patients´ initiation on ART.

*End time:* time when follow-up of patients was stopped. Free survival time: ADR-free time of patients after enrollment on ART. Anemia: non-anemic: hemoglobin level >10gram/deciliter, and Anemic; <10gram/deciliter.

## Results

**Socio-demographic and baseline characteristics study participants on initiation of ART:** in total, 592 HIV-infected adult patients were included in the study; 388 (65.5%) were females. Mean (+standard deviation) age of participants was 31.23 (+8.78) years, and 280 (47.3%) were aged 25-34 years (Table 1). In total, patients were followed for 2571py. Drug combinations used by patients in the study included D4T-3TC-NVP, AZT-3TC-NVP), D4T-3TC-EFV, AZT-3TC-EFV, TDF-3TC-NVP and TDF-3TC-EFV. Of the participants, 378 (64%) initiated on TDF based ART regimen combinations (Table 2). Of the participants, 295 (49.83%) and 482 (81.42%) accounted by participants had elementary school background and urban resident respectively (Table 1). Based on the WHO clinical stages, 319 (53.9%) of the participants were accounted by clinical III and IV (Table 2). Mean (+SD) of body mass index (BMI) and Median (interquartile range) of CD4 count of the study participants were 20.09 (+2.9) kilogram per square meter and 230 (142,322) cell per µL respectively. Among the participants, 587 (99.5%) participants screened for tuberculosis, 101 (17.2%) had tuberculosis infection (Table 2).

**Table 1:** socio-demographic characteristics of adult HIV-infected patients, enrolled on ART between 2006 and 2017 in government hospitals of Kaffa Zone, Ethiopia between July 2006 and August 2017

Baseline characteristics (N=592)	Number (%)
**Level of Hospitals**	
WMZMPH (primary)	82 (14%)
BSMGH (General)	510 (86%)
**Sex**	
Male	204 (34%)
Female	388 (66%)
**Age Category**	
15-24 years	115 (19%)
25-34 years	280 (47%)
>=35 years	197 (34%)
**Religion**	
Orthodox	436 (74%)
Protestant	60 (10%)
Muslim and Catholic	96 (16%)
**Marital status**	
Single	120 (20)
Married	332 (56%)
Divorced and widowed	140 (24%)
**Educational background**	
Illiterate	145 (24%)
Elementary	295 (50%)
Secondary and above	152 (26%)
**Residence**	
Rural	110 (19%)
Urban	482 (81%)

**Table 2:** initial clinical characteristics of adult HIV-infected patients, enrolled on ART between 2006 and 2017 in government hospitals of Kaffa Zone, Ethiopia between July 2006 and August 2017

Initial Clinical characteristics (N=592)	Number (%)
**Eligibility Criteria**	
Clinical	121 (20%)
CD4	243 (41%)
CD4+Clinical+TLC	228 (39%)
**WHO stage**	
I	158 (27%)
II	115 (19%)
III	268 (45%)
IV	51 (9%)
**Functionality status**	
Working	487 (82%)
Ambulatory	89 (15%)
Bed Ridden	16 (3%)
**BMI Category (Kg/M2)**	
18.5-24.9	394 (67%)
<18.5	168 (28%)
>=25	28 (5%)
**ART combinations**	
D4T-3TC-NVP	82 (14%)
AZT-3TC-NVP	45 (8%)
D4T-3TC-EFV	28 (5%)
AZT-3TC-EFV	59 (10%)
TDF based	378 (64%)
**CD4 Category in cell/microliter**	
<200	261 (44%)
>=200	336 (57%)
**Past Cotrimoxazole exposure**	
Yes	511 (86%)
No	81 (14)
**Active tuberculosis co-infection (N=587)**	
Yes	101 (17%)
No	491 (83%)
**Anti-TB treatment exposure**	
Yes	460 (78%)
No	132 (22%)

**Figure 1:**
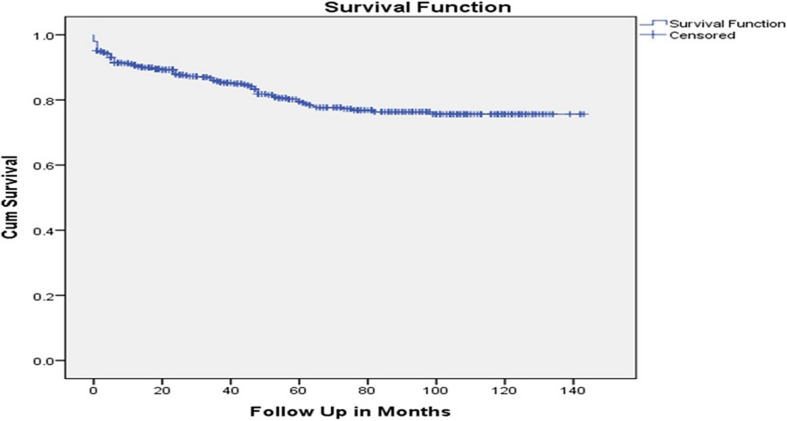
cumulative survival rates of ADR on HIV positive adult patients on HAART at government hospitals of Kaffa zone, Southern Ethiopia

**Figure 2:**
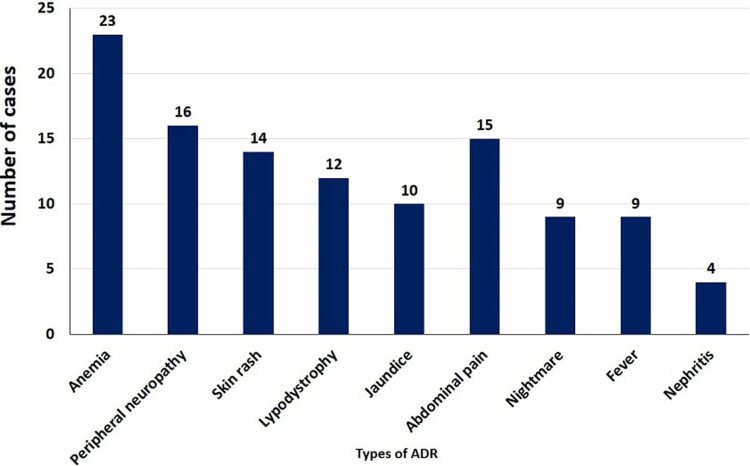
distribution of ADR types among HIV positive adults on HAART at Kaffa Zone Government Hospital, Southern Ethiopia, between July 2006 and August 2017

**Incidence of ADR:** after HIV-infected patients initiated on ART, 106 (18%) patients experienced at least 1 ADR. Incidence rate of ADR in this study was 4.1 per 100 py; (95%CI: 3.4, 5.0) with cumulative survival rate of 72%. The mean free survival time of the study participants were 115.65; (95%CI: 111, 120.3) months. The Kaplan Meier curve showed that hazard was sharply rising within 10 months of ART initiation, and continued to rise until approximately 80 months after initiation (Figure 1). In total, 112 ADRs were reported. Among those, 23 (20%) were anemia, and 16 (14%) were peripheral neuropathy (Figure 2).

**Predictors of ADR:** on bi-variable CRA, variables such as level of hospitals patients attended, marital status, educational background, religion, residence, eligibility criteria for patients to initiated on ART, WHO clinical stage at initiation, functionality status of the patient during enrollment on ART, ART combination, CD4 count category and tuberculosis co-infection had P< 0.25 and were included in multivariate CRA (Table 3, Table 3 suite).

**Table 3:** bi-variable and multivariable CRA of baseline and clinical characteristics for the determinants of the incidence of ADR among adult HIV-infected patients started ART in government hospitals of Kaffa Zone, Ethiopia between July 2006 and August 2017

Baseline and clinical characteristics	Survival status		CHR(95%CI)	AHR(95%CI
	Censored	Event(ADR)		
	No (%)	No (%)		
**Level of Hospitals**				
WMZMPH (primary)	64 (13.2%)	18 (17%)	1.5 (0.92,2.55)	1.61 (0.93,2.77)
BSMGH (General)	422 (86.6%)	88 (83%)	1	1
**Sex**				
Male	168 (34.6%)	36 (34%)	0.98 (0.65,1.46)	NS
Female	318 (65.4)	70 (66%)	1	
**Age Category**				
15-24 years	102 (21%)	13 (12.3%)	1.23 (0.47,3.24)	0.96 (0.48,1.93)
25-34 years	216 (44.4%)	64 (60.4%)	2.27 (0.98,5.25)	1.58 (0.992.50)
>=35 years	168 (34.6%)	29 (27.3%)	1	1
**Religion**				
Orthodox	363 (74.7%)	73 (68.9%)	0.62 (0.34,1.35)	0.62 (0.36,1.00)
Protestant	49 (10.1%)	11 (10.4%)	0.79 (0.33,1.90)	0.72 (0.34,1.53)
Muslim and Catholic	74 (15.2%)	22 (20.7%)	1	1
**Marital status**				
Single	92 (18.9%)	28 (26.4%)	1.45 (0.85,2.47)	1.60 (0.89,2.89)
Married	280 (57.6%)	52 (49.1%)	0.84 (0.53,1.35)	0.92 (0.60,1.50)
Divorced and widowed	114 (23.5%)	26 (24.5%)	1	1
**Educational background**				
Illiterate	121 (24.9%)	24 (22.6%)	1	1
Elementary	249 (51.2%)	46 (43.4%)	0.90 (0.55,1.48)	1.02 (0.61,1.71)
Secondary and above	116 (23.9%)	36 (34%)	1.41 (0.84,2.36)	1.67 (0.97,2.84)
**Residence**				
Rural	88 (18.1%)	22 (20.8%)	1.41 (0.88,2.25)	1.94 (1.18,3.20)*
Urban	398 (81.9%)	84 (79.2%)	1	1

* = statistically significant NS = Not Selected for multivariable analysis 1= Reference

**Table 3(suite):** bi-variable and multivariable CRA of baseline and clinical characteristics for the determinants of the incidence of ADR among adult HIV-infected patients started ART in government hospitals of Kaffa Zone, Ethiopia between July 2006 and August 2017

Baseline and clinical characteristics	Survival status		CHR(95%CI)	AHR(95%CI
	Censored	Event(ADR)		
	NO	NO		
**Eligibility Criteria**				
Clinical	104 (86%)	17 (14%)	0.77 (0.45,1.33)	NS
CD4	209 (86%)	34 (14%)	0.61 (0.40,0.94)	
CD4+Clinical+TLC	173 (76%)	55 (24%)	1	
**WHO stage**				
I	148 (30.5%)	10 (9.4%)	1	1
II	98 (20.2%)	17 (16%)	2.23 (1.02,4.87)	2.02 (0.91,4.46)
III	208 (42.8%)	60 (56.6%)	3.21 (1.64,6.27)	2.46 (1.22,4.95)*
IV	32 (6.6%)	19 (17.9%)	5.78 (2.69,12.43)	4.32 (1.88,9.93)*
**Functionality status**				
Working	414 (85.2%)	73 (68.9%)	1	1
Ambulatory	62 (12.8%)	27 (25.5%)	1.94 (1.25,3.02)	1.33 (0.80,2.18)
Bed Ridden	10 (2.1%)	6 (5.7%)	2.67 (1.16,6.14)	1.79 (0.74,4.31)
**BMI Category (Kg/M2)**				
18.5-24.9	331 (68.1%)	63 (59.4%)	1	
<18.5	131 (27.0%)	37 (34.9%)	1.28 (0.85,1.92)	NS
>=25	22 (4.6%)	6 (5.7%)	1.17 (0.51,2.71)	
**ART Category**				
D4T-3TC-NVP	58 (12.3%)	24 (20.8%)	2.33 (1.41,3.87)	1.78 (1.03,3.08)*
AZT-3TC-NVP	32 (6.6%)	13 (12.3%)	1.99 (1.12,3.55)	2.34 (1.20,4.57)*
D4T-3TC-EFV	16 (3.3%)	12 (11.3%)	2.06 (1.08,3.93)	2.86 (1.38,5.95)*
AZT-3TC-EFV	41 (8.4%)	18 (17%)	3.32 (1.74,6.31)	2.16 (1.21,3.90)*
TDF based	337 (69.3%)	41 (38.7%)	1	1
**CD4 Category in cell/microliter**				
<200	197 (39.5%)	64 (60.4%)	1	1
>=200	294 (60.5%)	42 (39.6%)	0.60 (0.39,0.86)	0.70 (0.44,1.01)
**Past Cotrimoxazole exposure**				
Yes	417 (85.8%)	94 (88.7%)	0.92 (0.50,1.69)	NS
No	69 (14.2%)	12 (11.3%)	1	
**Active tuberculosis co-infection**				
Yes	74 (15.4%)	27 (25.5%)	1.58 (1.02,2.44)	0.80 (0.46,1.38)
No	407 (84.6%)	79 (74.5%)	1	
**Exposure with anti-tuberculosis treatment**				
Yes	381 (78.4%)	79 (74.5%)	1.08 (0.69,1.67)	NS
No	105 (21.6%)	27 (25.5%)	1	

*= statistically significant NS=Not Selected for multivariable analysis **1=**Reference

Three variables remained significantly associated with ADR in multivariable analysis. The hazards of ADR among patients living in the rural areas were almost two times that of urban residents [AHR: 1.94(95% CI: 1.18, 3.20)]. On the bases of ART combination, the hazards of ADR were higher for persons taking D4T-3TC-NVP [AHR: 1.78 (95%CI: 1.03, 3.08)], AZT-3TC-NVP [AHR: 2.34 (95% CI: 1.20, 4.57)], D4T-3TC-EFV [AHR: 2.86 (95% CI: 1.38, 5.95)], and AZT-3TC-EFV [AHR: 2.16 (95%CI: 1.21,3.90)] compared with persons taking TDF-based regimens (TDF-3TC-NVP and TDF-3TC-EFV) regimens. In addition, hazards were higher for patients at WHO clinical stage III [AHR: 2.46 (95%CI: 1.22, 4.95)] and IV [AHR: 4.32 (95%CI: 1.88, 9.93)] than stage I (Table 3, Table 3 (suite)).

## Discussion

In our study of adverse drug reactions among HIV-infected persons, we found an overall incidence of 4.1 per 100 person-years of treatment, or approximately one in every twenty-five persons on treatment, per year. We found that rural persons were likelier to experience an ADR compared with urban persons, and that persons in later stages of AIDS were likelier to experience ADRs, compared with persons at earlier stages. In addition, hazards were lower for persons on TDF-based regimens than AZT and D4T based regimens. Risk was highest in the first five years on treatment, and gradually declined. Incidence of ADR in the current study was 4.1 per 100 py (95% CI: 3.4, 5.0), which was similar with findings from a study in Felege Hiwote referral hospital, Bahirdar, where the rate was 4.2 per 100 py (95% CI: 3.3, 5.4). However, this finding was inconsistent with findings from a study in the seven teaching hospital in Ethiopia; 9 per 100 py. The difference could be due to difference in study design, design of the previous study was a prospective cohort and data were collected objectively for research purpose.

Based on our finding, the risk of ADR among patients from rural areas were higher than patients from urban. Similarly, the proportion of ADR were higher at the rural on the studies conducted at Mali [8]. Even though studies were conducted in different setting in Ethiopia, residency was not significantly associated with ADR. The difference could be, patients in the rural may use traditional medicine and non-prescribed medicine.

In this study, the risk of ADR among D4T-3TC-NVP, AZT-3TC-NVP, D4T-3TC-EFV and AZT-3TC-EFV were higher than TDF base (TDF-3TC-NVP and TDF-3TC-EFV) combinations. This finding was similar with studies done in Duala Hospital; Cameroon, tertiary teaching hospital; Kenya and Debremarkose hospital; Ethiopia [6, 9, 10]. Additionally, similar result was observed on D4T based regimen in Hiwote Fana hospital Harer, Ethiopia [11]. In contrast incidence of ADR was higher in TDF/3TC/EFV in Jimma Hospital Ethiopia [12]. This could be due to small sample size in previous study, which was 233 and much smaller than our study. It is known that D4T base regimens were known to cause toxicity and removed from the treatment plan in different country including Ethiopia. Additionally, AZT based regimens were also known to cause ADR, but still it is given as an alternative in Ethiopia.

The risk of ADR among patients with WHO clinical stage III and IV were higher than WHO clinical stage I, this is similar with the studies conducted at Bahirdar, Ethiopia [13]. Similarly, in other study in Ethiopia; incidence of ADR was higher among patients on WHO clinical stage III. This could be due to drug interaction; patients on WHO clinical stage III and IV might be on other treatment with ART.

## Conclusion

The risk of ADR was much higher among adult HIV positive rural patients on ART. Additionally, adult HIV-infected patients within WHO clinical stage III and IV on HAART had higher risk of developing ADR than stage I. The study also concluded that patients on AZT and D4T based regimen had considerably higher risk of developing ADR than TDF based regimen. To reduce ADR, AZT-based regimens should not be given as a TDF alternative and increase TDF based regimens. ART providers should be prepared to see higher ADRs in rural and WHO clinical stage III/IV patients starting on ART, and give more attention. Additionally, we recommend further study on why incidence of ADRs were higher among rural patients than urban.

### What is known about this topic

- D4T as a regimen was declined from the treatment plan because of its higher incidence of ADR.

### What this study adds

- This study showed that AZT as a treatment is soul cause of ADR;
- The hazard of ADR among patients lived in the rural is much higher than patients lived in the urban;
- The hazard of ADR was higher among patients on clinical stage-3 and 4 than patients on clinical stage-1.

## References

[1] Fauci AS (2017). HIV/AIDS USA: U.S. department of health and human survice, National institute of health.

[2] World Health Organization (WHO) THE GLOBAL HEALTH OBSERVATORY.

[3] AIDS info (2017). Guidelines for the Use of Antiretroviral Agents in HIV-1-Infected Adults and Adolescents.

[4] Gudina EK, Teklu AM, Berhan A, Gebreegizabhier A, Seyoum T, Nega A (2017). Magnitude of Antiretroviral Drug Toxicity in Adult HIV patients in Ethiopia: A cohort study at seven teaching hospitals. Ethiop J Health Sci.

[5] Eluwa GI, Badru T, Akpoigbe KJ (2012). Adverse drug reactions to antiretroviral therapy (ARVs): incidence, type and risk factors in Nigeria. BMC Clin Pharmacol.

[6] Tadele A, Hiruy N, Shumye A (2016). Adverse Drug Reaction of Highly Active Antiretroviral Therapy on Adult Patients at Debre Markose Referal Hospital, Ethiopia: A Retrospective Study. Ethiop J Pub Hlth Nutr.

[7] Markose E, Worku A, Davi G (2008). Adherance to ART in PLWHA at Yirgalem Hospital, South Ethiopa. Ethiop J Health Dev.

[8] Oumar AA, Abdoulaye A, Maiga M, Sidibe Y, Cissoko Y, Konate I (2017). Adverse Drug reactionto Antiretroviral therapy (ART): Prospective study in HIV infected Adults in Sikasso(Mali). J Pharmacovigil.

[9] Luma HN, Doualla M-S, Choukem S-P, Temfack E, Ashuntantang G, Joko HA (2012). Adverse drug reactions of Highly Active Antiretroviral Therapy (HAART) in HIV infected patients at the General Hospital, Douala, Cameroon: a cross sectional study. Pan Afr Med J.

[10] Bhuvana KB, Hema NG, Sangeetha (2014). A prospective observetional study of Adverse drug reaction to antiretroviral therapy and risk factors in teritiary care teaching hospital. Int J Basic & Clin Pharmacolo.

[11] Woldegebreal F, Mitiku H, Teklemariam Z (2016). Magnitude of adverse drug reaction and associated factors among HIV infected adults on antiretroviral therapy in Hiwot Fana specialized University hospital, eastern Ethiopia. Pan Afr Med J.

[12] Tatiparthi R, Mamo Y (2014). Prevalence of ADRs and associated factors of antiretroviral treatment on HIV positive adults at Jush. Ind J Pharma Prac.

[13] Kindie E, Anteneh ZA, Worku E (2017). Time to Development of Adverse Drug reaction and associated factors among adult HIV positive patients on anti retroviral treatment in Bahirdar City, Northwest Ethiopia. PLoS One.

